# External and Internal Load Variables Encountered During Training and Games in Female Basketball Players According to Playing Level and Playing Position: A Systematic Review

**DOI:** 10.1186/s40798-022-00498-9

**Published:** 2022-08-19

**Authors:** Cody J. Power, Jordan L. Fox, Vincent J. Dalbo, Aaron T. Scanlan

**Affiliations:** 1 School of Health, Medical and Applied Sciences, Central Queensland University, Building 81/1.12, Bruce Highway, Rockhampton, QLD 4702 Australia; 2 Human Exercise and Training Laboratory, Central Queensland University, Rockhampton, QLD Australia; 3 Rural Clinical School, The University of Queensland, Rockhampton, QLD Australia

**Keywords:** Demands, Women, Monitoring, RPE, Microsensor, Video analysis, Heart rate, Methodological quality

## Abstract

**Background:**

Despite the growing global participation of females in basketball and number of studies conducted on the topic, no research has summarized the external and internal load variables encountered by female basketball players during training and games.

**Objective:**

To collate existing literature investigating external and internal load variables during training and games in female basketball players according to playing level (club, high-school, representative, collegiate, semi-professional, and professional) and playing position (backcourt and frontcourt players).

**Methods:**

A systematic review of the literature was performed using PubMed, SPORTDiscus, and Web of Science to identify studies published from database inception until June 11, 2021. Studies eligible for inclusion were observational and cross-sectional studies, published in English, reporting external and/or internal load variables during training sessions and/or games. Methodological quality and bias were assessed for each study prior to data extraction using a modified Downs and Black checklist. Weighted means according to playing level and playing position were calculated and compared if a load variable was reported across two or more player samples and were consistent regarding key methodological procedures including the seasonal phase monitored, minimum exposure time set for including player data (playing time during games), approach to measure session duration, and approach to measure session intensity.

**Results:**

The search yielded 5513 studies of which 1541 studies were duplicates. A further 3929 studies were excluded based on title and abstract review, with 11 more studies excluded based on full-text review. Consequently, 32 studies were included in our review. Due to the wide array of methodological approaches utilized across studies for examined variables, comparisons could only be made according to playing level for blood lactate concentration during games, revealing backcourt players experienced higher lactate responses than frontcourt players (5.2 ± 1.9 mmol·L^−1^ vs. 4.4 ± 1.8 mmol·L^−1^).

**Conclusions:**

Inconsistencies in the methods utilized to measure common load variables across studies limited our ability to report and compare typical external and internal loads during training and games according to playing level and position in female basketball players. It is essential that standardized methodological approaches are established for including player data as well as measuring session duration (e.g., total time, live time) and intensity (e.g., consistent rating of perceived exertion scales, intensity zone cut points) in future female basketball research to permit meaningful interpretation and comparisons of load monitoring data across studies.

## Key Points

- The wide assortment of load variables monitored and inconsistencies in the methods utilized to measure load variables across studies limit the ability to report and compare typical external and internal loads during training and games according to playing level and position in female basketball players.
- Standardized approaches are needed for categorizing playing level and position, deciding when to include player data in analyses (e.g., minimum exposure time), measuring session duration (e.g., total time, live time, session components), and measuring session intensity (e.g., consistent RPE scales, intensity zone cut points) in future female basketball research to permit meaningful interpretation and comparisons of load monitoring data across studies.
- Despite a rise in the popularity, professionalism, and number of studies reporting training and game loads in female basketball players, more longitudinal studies reporting load variables across various timeframes (e.g., sessional, daily, weekly, monthly, seasonal phase) and playing levels are needed to better understand the loading patterns experienced across the annual plan in female basketball teams.
- Backcourt female basketball players (guards) appear to experience higher BLa concentrations during games compared to frontcourt players (forwards and centers).

## Introduction

Basketball is one of the most popular team sports played among females, ranking second and fourth for participation in team sports among women in Australia [1] and New Zealand [2], respectively, and ranking second for participation in high-school athletes in the United States [3]. The strong participation base, growing audience, and increasing number of initiatives to promote and support female athletes [4] have led to a rise in professionalism in women’s basketball, bringing a more structured approach to training, game preparation, and recovery in teams using scientific concepts. A concept that has been increasingly applied in women’s basketball to optimize the training process is load monitoring. Load data can be categorized as external load representing the physical stimuli imposed on players during training and games or internal load representing the psycho-physiological responses of players to the physical stimuli encountered [5]. Given the practical merit of load monitoring, an increasing number of studies have quantified the loads encountered during training and games in various samples of female basketball players.

Load monitoring approaches in basketball are essential to inform and individualize the design of training programs and, in turn, optimize performance during competition while reducing the likelihood of maladaptive responses (e.g., illness, injury, or non-functional overreaching) in players [6, 7]. Specifically, when adequate training stimuli are applied, players experience positive improvements in the function of the targeted physiological systems, leading to improvements in performance [5, 8]. However, when excessive training stimuli are applied, players may fatigue [8, 9] resulting in reduced training tolerance and diminished performance [9, 10], increased risk of illness and injury [8], as well as an increased chance of cognitive and mood disturbances [8, 10]. Additionally, if training stimuli are inadequate, players may experience decay in fitness attributes, reducing performance (detraining) [5, 11]. Consequently, training is often periodized across seasonal phases of the annual plan with specific periods of functional overload or de-load [8, 9], which requires measurement of the external and internal loads encountered by players during training and games to ensure players are experiencing intended demands and responding favorably [8].

Evidence indicates linear growth in the number of publications quantifying the external and internal loads encountered during training and games in female basketball players across the past decade [12]. While several reviews have examined training [13, 14] and game loads [14–16] in basketball players, they have predominantly focused on male players. In this regard, only one review [15] has included female players, examining the activity demands and physiological responses encountered during basketball games in male and female players. There are established differences in biological attributes [17], biomechanical profiles [18], and contextual challenges (e.g., competition structure [shorter game durations in some leagues, number of games per season, time of day games are played] and finances allocated to tournaments) [19] between males and females. Consequently, evidence stemming from reviews focused on quantifying external and internal loads in male basketball players [14, 15] should not be simply applied to female basketball players. In turn, identifying the external and internal loads encountered during training and games in female basketball players is essential to permit evidence-driven training approaches, recovery plans, and player management strategies in female basketball players. Furthermore, given the varied physical attributes reported across playing levels [16] and playing positions [20] in basketball players, the external and internal loads experienced by players should be examined according to playing level and position for greater specificity in the evidence provided.

A systematic analysis of the literature quantifying training and game loads in female basketball players is necessary for several reasons. (1) More research quantifying game loads in female basketball players has been conducted since the previous review, which considered studies published until September 2016 [15]. (2) The previous review [15] only examined game loads; therefore, no literature has synthesized original research quantifying external and internal loads encountered during training in female basketball players. (3) External load variables reported in the previous review [15] were limited to frequencies, distances, and durations of various basketball-specific activities measured via video-based time motion analysis (TMA). However, other technologies such as microsensors and local positioning systems (LPS) have become more prominent for objective measurement of external load in female basketball since the previous review [15]. (4) Male and female players were examined in combination when assessing differences between playing level and playing position in the previous review [15]. Consequently, the aim of our systematic review was to collate published data quantifying the external and internal loads encountered by female basketball players during training and games according to playing level and playing position.

## Methods

### Search Strategy

Studies were identified via PubMed (MEDLINE), Web of Science, and SPORTDiscus using the following search terms: training, competition, games, work, intensit*, load, demands, exertion, physical, RPE, SHRZ, TRIMP, ‘heart rate,’ HR, ‘micromechanical electrical system,’ MEMS, micro*, IMU, ‘inertial movement analysis,’ IMA, accelerat*, decelerat*, accelerome*, ‘inertial measurement unit,’ ‘local positioning system,’ LPS, ‘ultra-wide band,’ UWB, ‘radio frequency identification,’ RFID, PlayerLoad, ‘repeated high intensity effort,’ RHIE AND women, female AND basketball. Search terms relating to load were joined using the OR operator, then combined with (women OR female) AND basketball. Terms which have various grammatical suffixes were indicated using ‘*’. All searches were conducted using ‘all fields.’ Our search terms were developed to consider research studies published online or in-print from database inception until June 11, 2021.

### Selection Criteria

The process for screening studies followed the 2020 Preferred Reporting Items for Systematic Reviews and Meta-Analyses (PRISMA) guidelines [21]. Our review was not registered with PROSPERO. Studies considered for inclusion in our review were original peer-reviewed studies published in English that reported external and/or internal load variables during training and/or games in female basketball players. In this regard, no restrictions were placed on how external or internal load variables were tabulated (e.g., individual training session vs. the sum of all training sessions in a week) or on the player sample (i.e., age, playing level, or playing experience). Our review was restricted to cross-sectional and/or longitudinal observational study designs given experimental studies implementing an intervention may have influenced the typical loads experienced by players during training and/or games. In addition, studies examining wheelchair basketball players were excluded from our review given they may have led to inaccurate summations of data across populations due to the unique external and internal loads encountered during wheelchair basketball [22].

In our review, load was categorized as external load or internal load. External load was defined as the physical stimuli imposed on players during training and games, while internal load was defined as the psycho-physiological reactions of players to the physical stimuli encountered [5]. Given exposure is a measure of duration and does not objectively quantify the external demands or intensity of training sessions or games, studies using exposure as the only external load variable were excluded from our review.

**Fig. 1:**
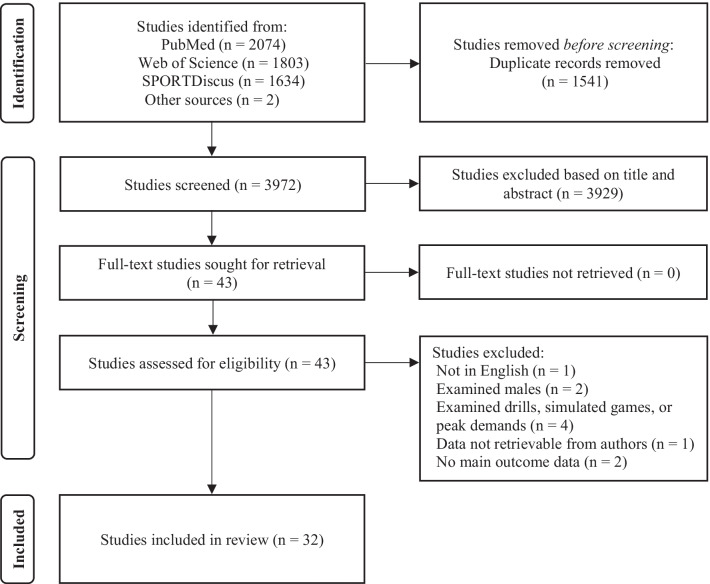
Preferred Reporting Items for Systematic Reviews and Meta-Analysis (PRISMA) flow diagram of search strategy

The process for screening studies included in our review is shown in Fig. 1. Following the elimination of duplicates, the abstracts of all studies identified in the search were screened independently against the pre-defined selection criteria by two authors (C.J.P. and J.L.F.). Any disagreements between the two authors regarding study inclusion were further discussed and, if agreement was not reached, a third author (A.T.S.) was consulted to establish consensus. Following the screening of title and abstract, the full-text version of the remaining studies was then obtained and independently screened by the same two authors to determine eligibility. Any disagreements between the two authors regarding study inclusion were again discussed and, if agreement was not reached, a third author was consulted to establish consensus. The reference lists of included studies following screening of full-text versions were then reviewed to identify any potential studies not captured in the original search.

### Assessment of Methodological Quality and Bias

A modified version (Table 1) of the Downs and Black checklist [23] was utilized to conduct methodological quality and risk-of-bias assessments. The modified 11-item checklist (Table 1) was chosen as it is valid [23] and has been used to assess observational studies examining external and internal loads in team sports [13, 24, 25]. The modified Downs and Black quality assessment tool consisted of 3 sections which assessed the quality of reporting the results, external validity, and internal validity-bias. The maximum total score was 11, with a higher total score indicating a higher quality of evidence for the specific study. The quality and bias assessment was carried out by two authors (C.J.P. and J.L.F.). Any disagreement in the outcome of the appraisal was discussed and a third author (A.T.S.) consulted if consensus could not be reached. Each item was scored as ‘1’ (yes) or ‘0’ (no/unable to determine), with each of the 11 items summed to provide the total score.

**Table 1:** Modified Downs and Black checklist used to assess methodological quality of the included studies

Question number	Question
	*Reporting*
1	Is the hypothesis/aim/objective of the study clearly described?
2	Are the main outcomes to be measured clearly described in the Introduction or Methods section?
3	Are the characteristics of the patients included in the study clearly described?
4	Are the main findings of the study clearly described?
5	Does the study provide estimates of the random variability in the data for the main outcomes?
6	Have actual probability values been reported (e.g., 0.035 rather than < 0.05) for the main outcomes except where the probability value is less than 0.001?
	*External validity*
7	Were the subjects asked to participate in the study representative of the entire population from which they were recruited?
8	Were those subjects who were prepared to participate representative of the entire population from which they were recruited?
	*Internal validity-bias*
9	If any of the results of the study were based on ‘data dredging,’ was this made clear?
10	Were the statistical tests used to assess the main outcomes appropriate?
11	Were the main outcome measures accurate (valid and reliable)?

### Data Extraction

Data were extracted from each study by the lead author (C.J.P.), with all co-authors reviewing extracted data for accuracy and completeness. Player characteristics and outcome variables are reported as mean ± standard deviation (SD) where available. If studies only presented data in figures, attempts were made to contact the authors via email for access to the numerical data. If the contacted authors were unable to provide the required data, means were retrieved from figures using WebPlotDigitizer (Edition 4.3, Austin, TX, USA). WebPlotDigitizer is a semiautomatic, open source, web-based tool with acceptable validity [26] and reliability [26, 27] for extracting numerical data from figures. If SD were not reported in relevant figures and could not be obtained, these values were identified as ‘not provided (NP)’ in text and only means were reported in these instances. The following data were extracted from each study, where reported:

- Player characteristics—playing level, geographical location, sample size, age (years), body mass (kg), stature (cm), and playing experience (years).
- Outcome variables
  o External load variables—frequency (*n*), distance covered (m), and duration (%) performing basketball-specific activities identified based on movement type and/or intensity, accumulated load (reported as player load or PlayerLoad™ [PL]; arbitrary units [AU]), average net force (N), external training impulse (TRIMP; AU), and speed (m·s^−1^). Inertial movement analysis variables were reported as total accelerations (*n*), decelerations (*n*), jumps (*n*), and high-intensity events (accelerations, decelerations, changes of direction, and jumps; *n*). Definitions and criteria used to measure external load variables across studies included in this review are detailed in Table 2.
  p Internal load variables—absolute (beats·min^−1^) and relative (percentage of peak HR; %HR_peak_) HR responses, blood lactate concentration (BLa; mmol·L^−1^), internal TRIMP (AU) (calculated using various methods stipulated in Table 2), rating of perceived exertion (RPE; AU), and session-RPE load (sRPE) (individualized RPE multiplied by session duration in minutes [28]; AU). Absolute and relative HR was reported according to live and total playing time during games (defined in Table 3).

**Table 2:** Categories and definitions of external load variables and internal training impulse (TRIMP) variables in the included studies

Load variable	Definition
*Activity frequency, duration, and distance covered*	
Standing/walking	Activity of no greater intensity than walking. No distinction was made between different intensities of walking [29–31] OR Multidirectional movement performed at 0–1 m∙s^−1^, when not in a defensive stance [32]. A distinction between standing and walking was made in one study whereby standing was identified as movement performed at < 1 m∙s^−1^ and walking was identified as movement performed at 1.00–1.81 m∙s^−1^ [33]
Jogging or low-speed running	Forwards or backwards activity without urgency but at a greater intensity than walking [29–31] OR Multidirectional movement performed at 1.1–3.0 m·s^−1^, when not in a defensive stance [32] OR Forwards or backwards movement at 1.81–2.83 m·s^−1^ [33]
Running or moderate-speed running	Forwards or backwards activity at an intensity greater than jogging with a moderate degree of urgency, but not approaching an intense level of movement [29–31] OR Multidirectional movement performed at 3.1–7.0 m·s^−1^, when not in a defensive stance [32] OR Forward or backwards movement at 2.83–4.00 m·s^−1^ [33]
Sprinting or maximal-speed running	Forwards movement at an intensity greater than running, characterized by elongated strides, effort and purpose at or close to maximum [29–31] OR Multidirectional movement performed at > 7.0 m·s^−1^, when not in a defensive stance [32] OR Forward or backwards movement at > 4 m·s^−1^ [33] or > 5.8 m·s^−1^ [34]
Low-intensity shuffling or specific movements	Movement without urgency in a sideways or backwards direction using a shuffling action of the feet [29, 30] OR Movement performed strictly in a defensive stance at ≤ 2 m·s^−1^ [32] OR Any foot action that differed from ordinary walking or running at < 1.67 m·s^−1^ [31]
Moderate-intensity shuffling or specific movements	Movement at a medium intensity with a moderate degree of urgency in a sideways or backwards direction using a shuffling action of the feet [29, 30] OR Any foot action that differed from ordinary walking or running at 1.67–2.50 m·s^−1^ [31]
High-intensity shuffling or specific movements	Movement at a high intensity characterized by effort and urgency in a sideways or backwards direction using a shuffling action of the feet [29, 30] OR Multidirectional movement performed strictly in a defensive stance at > 2 m·s^−1^ [32] OR Any foot action that differed from ordinary walking or running at > 2.5 m·s^−1^ [31]
Jumping	The time from the initiation of the jump motion until the landing is complete [29, 30] OR Any movement whereby a player initiates a jump and breaks feet contact with the ground [32] OR Any movement which involves jumping from the ground with an impulse involving more than 400 ms of flight time, to land in the same or another place [35] OR Calculated using a proprietary, undisclosed algorithm [36]
Dribble	Movement in which a player is actively in possession of and dribbling the ball [32]
Upper body	Movement that involves raising one or both arms above the horizontal plane at the level of the shoulder [32]
Steps	Movement that implies advancing with a flight time of < 400 ms [35, 37]
*Inertial movement analysis (IMA) variables*	
High-intensity IMA events	The sum of accelerations (− 45° to 45°; where 0° is forward), decelerations (− 135° to 135°), and changes of direction (− 135° to − 45° for left and 45° to 135° for right) at ≥ 3.5 m·s^−1^ [38, 36]
*Accelerometer-derived variables*	
PlayerLoad™	A proprietary metric sampled at 100 Hz and calculated as the square root of the sum of the squared rate of change in acceleration across the transverse (x), coronal (y), and sagittal (z) planes multiplied by a scaling factor of 0.01 [38, 36]: $Player load^{{{\text{TM}}}} = \left[ {\left( {\surd Ac1_{n} - Ac1_{n - 1} } \right)^{2} + \left( {Ac2_{n} - Ac2_{n - 1} } \right)^{2} + \left( {Ac3_{n} - Ac3_{n - 1} )^{2} } \right)} \right] * 0.01$
Player load	The vectorial magnitude derived from the triaxial accelerometer, sampling at 100 Hz and using the formula [35, 37]: $Player load_{t = n} \mathop \sum \limits_{t = 0}^{t = n} \surd (Z_{t = i + 1} - Z_{t = i} )^{2} + (X_{t = i + 1} - X_{t = i} )^{2} + (Y_{t = i + 1} - Y_{t = i} )^{2}$ OR Derived from the triaxial accelerometer sampling at 100 Hz or ultra-wide band antennae sampling at 20 Hz, and calculated using the formula [34]: $Player load_{n} = \surd [(ACx_{n} - Acx_{n - 1} )^{2} + (ACy_{n} - Acy_{n - 1} )^{2} + \left( {ACz_{n} - ACz_{n - 1} )^{2} } \right]/100$
TRIMP	The product of PlayerLoad™·min^−1^ and session duration [39]
Average net force (AvF_net_)	The three planes of triaxial accelerations are filtered using a dual-pass, fourth-order Butterworth filter (high pass: 0.1 Hz, low pass: 15 Hz). After filtering, the product of the instantaneous acceleration vector and player’s body mass are used to determine instantaneous net force [40, 41]
*Internal training impulse (TRIMP) variables*	
Edwards’ Summated-Heart-Rate-Zones	Multiply the time spent (min) in five different heart rate zones by the corresponding weighting factor for each zone (50–60% HR_max_ = 1; 60–70% HR_max_ = 2; 70–80% HR_max_ = 3; 80–90% HR_max_ = 4; and 90–100% HR_max_ = 5), then sum the calculated values [42]
Modified Summated-Heart-Rate-Zones	Multiply the time spent (min) in five different heart rate zones by the corresponding weighting factor for each zone (50–60% HR_peak_ = 1, 60–70% HR_peak_ = 2, 70–76% HR_peak_ = 3, 77–84% HR_peak_ = 4, and 85–100% HR_peak_ = 5), then sum the calculated values [43] OR Multiply the time spent (min) in five different heart rate zones by the corresponding weighting factor for each zone (50–59.9% HR_max_ = 1, 60–69.9% HR_max_ = 2, 70–79.9% HR_max_ = 3, 80–89.9% HR_max_ = 4, and 90–100% HR_max_), then sum the calculated values [44]
Banister’s TRIMP	Banister’s TRIMP = D × (Δ HR ratio) × e^(b × Δ heart rate ratio)^, where D = session duration (min), e = constant set at 2.718, b = weighting factor set at 1.67 for females, and Δ HR ratio = (average heart rate during exercise − resting heart rate) ÷ (maximal heart rate during exercise − resting heart rate) [45]

**Table 3:** Definitions of methods for measuring training or game duration

Method	Studies
Training	
Start to the end of training inclusive of warm-up/down	[40, 46, 41, 47]
Start to the end of training excluding stretching exercises	[48]
Start to the end of training excluding warm-up only	[45]
Start to the end of training excluding warm-down only	[49]
Did not report how training duration was determined	[35, 50, 51, 38, 44, 50–54]
Games	
* Live time*	
All instances when the clock was running	[34]
All moments when the clock was running and players were on the court, inclusive of short moments in which the clock was stopped but the ball was live, and players were active during in-bound passes	[29–32]
When the player was actively participating in the game and the timer was running	[55]
Time on the court, excluding time-outs	[56]
*Total time*	
Game time excluding half-time and quarter breaks as well as time-outs	[57]
All instances that a player was on the court, including stoppages in play, but excluding inter-quarter breaks and time during which the player was substituted out of the game	[32, 58, 37, 33]
Game time including all stoppages except time-outs, quarter-time breaks and half-time breaks	[35, 36]
Game time including all stoppages, time-outs, and inter-quarter breaks	[59, 41]
Game time excluding the warm-up but including rest periods	[48]
Game time including all stoppages except quarter and half-time breaks	[43]
Did not report how game duration was determined	[60]

### Data Analysis

Extracted data were reported as mean ± SD. Where possible, a sample mean ± SD was reported for each study. Furthermore, extracted data were reported according to playing level, which was categorized from lowest to highest as: club, high-school, collegiate (i.e., college and university players), representative (i.e., trained athletes selected into a representative team), semi-professional (i.e., some players are full-time/contracted athletes), or professional (i.e., all players are full-time, contracted athletes). Where possible, extracted data were also reported according to playing position which was categorized as backcourt (i.e., point guards and shooting guards) or frontcourt (i.e., small forwards, power forwards, and centers) players. The grouping of players into backcourt and frontcourt players has been commonly adopted in past research and accounts for players transitioning between positions during different phases of play [32, 40, 61, 62]. If studies reported playing position data as guards, forwards, and centers or point guards, shooting guards, small forwards, power forwards, and centers, the reported values were recalculated and grouped according to the current definition of backcourt and frontcourt playing positions. If the same outcome variable (e.g., PL) for a specific playing level and/or playing position was reported in more than one player sample (within the same study or across separate studies), weighted means and SD were calculated using a free, online-based tool [63]. Conclusions regarding differences in external and internal loads according to playing level and position for specific variables were made where values were reported for two or more player samples within the same playing level or the same playing position. Furthermore, weighted means and SD were only calculated and compared if key methodological procedures were consistent across player samples (within the same study or across separate studies) including the seasonal phase monitored, minimum exposure time set for including player data (i.e., playing time during games), approach to measure session duration (see Table 3 for approaches adopted in the literature), and approach to measure session intensity (e.g., type of RPE scale, method to identify HR_peak_, intensity zone cut point values).

## Results

### Study Selection and Methodological Quality

A total of 5511 studies were identified in the original search. Two additional studies [57, 58] not identified in the search, but known to the authors, were labeled as potentially relevant bringing the total to 5513 studies. Subsequently, 1541 duplicate studies were removed and a further 3929 studies were excluded based on title and abstract. As a result, 43 full-text studies were screened with 11 studies being removed, leaving 32 studies included in our review. The full results of the search are presented in Fig. 1. Methodological quality and bias scores ranged from 6 to 11 out of 11 (mean ± SD: 9 ± 1) and are presented in Table 4. No studies were excluded based on methodological quality or bias.

**Table 4:** Results of methodological quality assessment for included studies

Study	Downs and black checklist question number											Total
	Reporting					External validity		Internal validity-bias				
	1	2	3	4	5	6	7	8	9	10	11	
Anderson et al. [51]	1	1	0	1	0	0	0	0	1	1	1	6
Conte et al. [30]	1	1	1	1	1	0	0	1	1	1	1	9
Coyne et al. [39]	1	1	1	1	1	1	0	0	1	1	0	8
Cruz et al. [52]	1	1	1	1	1	0	0	0	1	0	1	7
Delextrat et al. [31]	1	1	1	1	1	1	1	1	1	1	1	11
Ghali et al. [50]	1	1	1	1	1	1	1	1	1	1	1	11
Kraft et al. [44]	1	1	0	1	1	0	1	1	1	1	1	9
Lastella et al. [46]	1	1	1	1	1	1	0	0	1	1	1	9
Lukonaitienė et al. [45]	1	1	1	1	1	1	1	1	1	1	1	11
Lupo et al. [42]	1	1	1	1	1	1	0	0	1	1	1	9
Matthew and Delextrat [29]	1	0	1	1	1	0	0	0	1	1	1	7
Nunes et al. [53]	1	1	1	1	1	0	0	0	1	1	1	8
Oba and Okuda [57]	1	0	0	1	1	0	0	0	1	1	1	6
Otaegi and Los Arcos [48]	1	1	1	1	1	0	0	1	1	1	1	9
Palmer et al. [41]	1	1	1	1	1	0	1	0	1	1	1	9
Paulauskas et al. [47]	1	1	1	1	1	1	1	1	1	1	1	11
Peterson and Quiggle [38]	1	1	1	1	1	1	0	0	1	1	1	9
Piedra et al. [54]	0	1	1	1	1	1	0	0	1	1	1	8
Portes et al. [34]	1	1	1	1	1	1	1	0	1	1	1	10
Ransdell et al. [36]	1	1	0	1	1	1	1	1	1	1	1	10
Reina et al. [35]	1	1	1	1	0	1	0	1	1	1	0	8
Reina et al. [60]	1	1	1	1	1	1	1	0	1	1	1	10
Reina et al. [37]	1	1	1	1	1	0	0	0	1	1	0	7
Reina et al. [33]	1	1	0	1	0	1	1	0	1	1	0	7
Rodriguez-Alonso et al. [56]	1	0	1	1	1	0	0	0	1	1	1	7
Sanders et al. [43]	1	1	0	1	1	1	0	0	1	1	1	8
Sanders et al. [59]	1	1	0	1	1	1	0	0	1	1	1	8
Sansone et al. [49]	1	1	1	1	1	1	0	0	1	1	1	9
Scanlan et al. [32]	1	1	1	1	1	1	0	0	1	1	1	9
Staunton et al. [40]	1	1	1	1	1	1	0	0	1	1	1	9
Vala et al. [55]	1	1	1	1	1	1	0	0	1	1	1	9
Vencúrik and Nykodým [58]	1	1	1	1	1	0	0	0	1	1	1	8

1 = yes; 0 = no/unable to determine

### Player Characteristics and Methodological Approaches

The characteristics of players recruited and key methodological approaches adopted (i.e., season phase, monitoring period duration, monitoring method, and equipment used) in the included studies are presented in Table 5. Sample sizes across studies ranged from 6 to 48 players. The mean age of players ranged from 13 to 27 years, with players competing across various playing levels, including club [35, 37, 48, 50], high-school [57], collegiate [29, 36, 38, 43, 44, 51, 57, 59], representative [33, 34, 42, 45, 46, 52, 60], semi-professional [32, 41, 49], and professional [30, 31, 39–41, 47, 53–58] competitions. Studies monitored players across different seasonal phases including the pre-season [54], the in-season [29–32, 35–38, 40, 41, 43, 47–50, 52, 54–56, 59], playoffs [57], training camps [39, 42, 45, 46, 53], and tournaments [33, 34, 60], with some studies not specifying the seasonal phase monitored [44, 51, 58]. The monitoring period durations also varied across studies with the number of weeks monitored ranging from 1 [50] to 32 [54] weeks (mean ± SD: 12 ± 9 weeks), and the number of games monitored ranging from 1 [57] to 166 [36] (mean ± SD: 19 ± 38 games). A range of different monitoring methods were used to measure external and internal load variables across studies (i.e., video-based TMA, microsensors, LPS, sRPE, HR, and BLa). Approaches to measure specific load variables with the same monitoring method also varied across some studies. For instance, different RPE scales were adopted to measure sRPE (Foster’s scale [46, 48, 49, 51, 53] or Borg’s category-ratio (CR10) scale [47, 52, 54]), with some studies not specifying the RPE scale used [39, 44].

**Table 5:** Participant characteristics and key methodological approaches from each study included in our systematic review

Study	Playing level (Country)	Sample size	Age (years)	Stature (cm)	Body mass (kg)	Seasonal phase (duration)	Monitoring method (equipment)
Anderson et al. [51]	Collegiate (USA)	12	20 ± 3	–	–	- (20 weeks)	Session-RPE load (Foster’s scale)
Conte et al. [30]	Professional (Italy)^a^	12	27 ± 4	184 ± 9	77.5 ± 15.1	In-season (5 games)	Video TMA (SONY HDR-CX115)
Coyne et al. [39]	Professional (Unknown)^a^	13	29 ± 4	186 ± 9.8	77.9 ± 11.6	Training camp (18 weeks)^b^	Microsensor (Catapult) Session-RPE load (-)
Cruz et al. [52]	Representative (Spain)^a^	10	17.2 ± 0.4	177.2 ± 9.5	71.8 ± 15.0	In-season (9 weeks)	Session-RPE load (Borg’s CR-10 scale)
Delextrat et al. [31]	Professional (Spain)^a^	42	25.9 ± 4.3	183.4 ± 9.0	–	In-season (3 games)	Video TMA (-)
Ghali et al. [50]	Club level (Canada)^a^	60	–	–	–	In-season (1 week)	Microsensor (VERT 2.0) Session-RPE load (Foster’s scale)
Kraft et al. [44]	Collegiate (USA)	–	–	–	–	- (124 sessions)	Session-RPE load (-) HR (Polar H7)
Lastella et al. [46]	Representative (Australia)^a^	11	17.3 ± 0.9	182.3 ± 5.5	77.0 ± 7.2	Training camp (118 sessions)	Session-RPE load (Foster’s scale)
Lukonaitienė et al. [45]	Representative, Under-18 (Lithuania)^a^	12	18.0 ± 0.5	180.4 ± 7.5	72.7 ± 9.3	Training camp (3 weeks)	Microsensor (Catapult OptimEye s5) Session-RPE load (Borg’s CR-10 scale) HR (Polar H10)
	Representative, Under-20 (Lithuania)^a^	12	19.6 ± 0.8	178.6 ± 6.4	68.0 ± 5.9		
Lupo et al. [42]	Representative (Italy)^a^	15	16.7 ± 0.5	178 ± 9	72 ± 9	Training camp (15 sessions)	HR (Polar H7)
Matthew and Delextrat [29]	Collegiate (United Kingdom)^a^	9	25.8 ± 2.5	173 ± 5	63.2 ± 4.5	In-season (9 games)	Video TMA (JVC- × 400) HR (Polar S810) BLa (Analox LM5 analyzer)
Nunes et al. [53]	Professional (Brazil)^a^	19	26 ± 5	181.8 ± 8.2	75.6 ± 12.6	Training camp (12 weeks)^b^	Session-RPE load (Foster’s scale)
Oba and Okuda [57]	High-school, Collegiate, and Professional (Japan)^a^	–	–	–	–	Playoffs (3 games)	Video TMA (DKH Co. PTS-110)
Otaegi and Los Arcos [48]	Club level, Under-15 (Spain)^a^	8	14.9 ± 0.6	161 ± 1	58.2 ± 7.6	In-season (24 sessions, 7 games)	Session-RPE load (Foster’s scale)
	Club level, Under-16 (Spain)^a^	11	15.1 ± 0.7	164 ± 1	62.8 ± 7.2	In-season (26 sessions, 8 games)	
Palmer et al. [41]	Semi-professional (Australia)	12	28.1 ± 5	176 ± 9.7	75.9 ± 18.2	In-season (33 sessions, 21 games)	Microsensor (ActiGraph GT9X)
	Professional (Australia)	12	25.2 ± 5.9	180.6 ± 10.7	79.3 ± 17.1	In-season (54 sessions, 20 games)	Microsensor (ActiGraph GT9X)
Paulauskas et al. [47]	Professional (Lithuanian)^a^	29	21 ± 5	181 ± 7	71 ± 7	In-season (24 weeks)	Session-RPE load (Borg’s CR-10 scale)
Peterson and Quiggle [38]	Collegiate (USA)	5	20 ± 1.0	178 ± 14	–	Pre-season and in-season (20 weeks)	Microsensor (Catapult OptimEye s5)
Piedra et al. [54]	Professional (Spain)	11	23.4 ± 3	182.2 ± 9.6	78.6 ± 13.9	Pre-season and in-season (32 weeks)	Session-RPE load (Borg’s CR-10 scale)
Portes et al. [34]	Representative (Spain)^a^	48	17 ± 1	176 ± 7	67.2 ± 6.2	Playoffs (3 games)	LPS (WIMU Pro)
Ransdell et al. [36]	Collegiate (USA)^a^	6	19.7 ± 1.5	–	–	In-season (144 games)	Microsensor (Catapult OptimEye s5)
Reina et al. [35]	Club level (Spain)^a^	12	–	163 ± 6	56.7 ± 6.6	In-season (35 sessions, 8 games)	LPS (WIMU Pro) HR (Garmin)
Reina et al. [60]	Representative (Spain)^a^	G = 13	–	168.6 ± 5.9	–	Playoffs (3 games)	LPS (WIMU Pro)
		F = 22	–	176.9 ± 6.0	–		
		C = 13	–	183.8 ± 4.7	–		
Reina et al. [37]	Club level (Spain)^a^	10	21.7 ± 3.6	168.5 ± 3.6	59.5 ± 12.3	In-season (22 sessions, 8 games)	LPS (WIMU Pro) HR (Garmin)
Reina et al. [33]	Representative (Spain)^a^	G = 13	–	168.6 ± 5.9	–	Playoffs (6 games)	LPS (WIMU Pro)
		F = 22	–	176.9 ± 6.0	–		
		C = 13	–	183.8 ± 4.7	–		
Rodriguez-Alonso et al. [56]	Professional, Olympic (Spain)^a^	14	25.8 ± 2.1	180.9 ± 8.0	71.7 ± 7.6	In-season (7 games)	HR (Sport-tester 4.000) BLa (GM7 micro-stat analyzer)
	Professional, Division I (Spain)	11	19.3 ± 2.8	175.1 ± 6.5	71.9 ± 8.7	In-season (3 games)	
Sanders et al. [43]	Collegiate (USA)	G = 3	20.3 ± 1.2	172.7 ± 2.5	72.6 ± 3.4	In-season (31 games)	HR (Polar Team)
		F = 3	20.0 ± 1.7	181.2 ± 1.5	80.8 ± 4.1		
		C = 4	19.3 ± 1.3	182.2 ± 6.7	80.3 ± 6.0		
Sanders et al. [59]	Collegiate (USA)	11	19.6 ± 1.4	179.7 ± 6.0	78.5 ± 5.7	In-season (31 games)	HR (Polar Team)
Sansone et al. [49]	Semi-professional (Italy)	13	22 ± 3	171.7 ± 6.3	66.3 ± 7.0	In-season (14 weeks)	Session-RPE (Foster’s scale)
Scanlan et al. [32]	Semi-professional (Australia)^a^	12	22.0 ± 3.7	174.2 ± 6.9	72.9 ± 14.2	In-season (8 games)	Video TMA (Basler A602FC) HR (Polar Team) BLa (Accusport analyzer)
Staunton et al. [40]	Professional (Australia)	9	27 ± 5	182 ± 8	81 ± 12	In-season (18 sessions)	Microsensor (ActiGraph GT9X)
Vala et al. [55]	Professional, Division I (Czech Republic)^a^	8	22.7 ± 1.9	176.6 ± 7.9	68.9 ± 6.4	In-season (8 games)	HR (Polar Team)
	Professional, Division II (Czech Republic)^a^	9	24.1 ± 2.3	179.1 ± 8.4	71.7 ± 10.5	In-season (8 games)	HR (Polar Team)
Vencúrik and Nykodým [58]	Professional (Czech Republic)^a^	8	20 ± 3	179.9 ± 4.5	66.8 ± 5.3	- (2 games)	HR (Suunto Team)

*USA* United States of America, *Endash (–)* not reported, *RPE* Rating of perceived exertion, *TMA* Time-motion analysis, *CR-10* Category-ratio 10, *HR* Heart rate, *LPS* Local position system, *G* Guards, *F* Forwards, *C* Centers, *BLa* Blood lactate concentration, ^a^Player samples that were recategorized by the authors into club, high-school, collegiate, representative (trained athletes selected into a representative team), semi-professional (some players are full-time/contracted athletes), or professional (all players are full-time/contracted athletes) playing levels, ^b^Players were monitored leading into an international tournament

### External and Internal Loads During Training

#### Individual Training Sessions

The external and internal loads experienced during basketball training reported in female players are shown in Table 6. Average net force and sRPE were the only variables reported in individual training sessions across multiple studies. Average net force was reported in multiple studies examining professional players and ranged from 272 ± NP N [41] to 293 ± 40 N [40] with a weighted mean of 281 ± NP N across studies [40, 41]. sRPE ranged from 213 ± 54 AU in club players [48] to 711 ± 282 AU in collegiate players [44]. sRPE was reported in multiple studies examining club players, ranging from 213 ± 54 AU [48] to 235 ± 39 AU [48] with a weighted mean of 226 ± 46 AU [48], as well as collegiate players, ranging from 530 ± NP AU [51] to 711 ± 282 AU [44]. A weighted mean could not be calculated for sRPE in collegiate players given the seasonal phase monitored, approach to measure session duration, and adopted RPE scale were not clearly identified across studies.

**Table 6:** Individual training session, total daily training (if more than one training session), weekly training only, and weekly training and games combined external and internal load variables experienced by female basketball players according to playing position and playing level

Study	Playing level	Sample	External load variables								
			Individual training sessions and total daily training load						Weekly training and game load		
			AvF_net_ (N)	PL (AU)	Jumps (n)	Jumps (n·min^−1^)	Steps (n)	Steps (n·min^−1^)	PL (AU)	High-intensity IMA events (n)	TRIMP (AU)
Coyne et al. [39]	Professional	All players	–	–	–	–	–	–	–	–	2787 ± 772
Lukonaitienė et al. [45]	Representative	All players—U18	–	816 ± 333^a^	–	–	–	–	–	–	–
		All players—U20	–	706 ± 295^a^	–	–	–	–	–	–	–
Palmer et al. [41]	Semi-professional	All players	280 ± NP	–	–	–	–	–	–	–	–
	Professional	All players	272 ± NP	–	–	–	–	–	–	–	–
Peterson and Quiggle [38]	Collegiate	All players	–	–	–	–	–	–	4073 ± 900	959 ± 228	–
Reina et al. [35]	Club	All players	–	38 ± 4^b^	48.2 ± NP	0.65 ± NP	1716 ± NP	22.9 ± NP	–	–	–
Staunton et al. [40]	Professional	All players	293 ± 40	–	–	–	–	–	–	–	–

Study	Playing level	Sample	Internal load variables						
			Individual training sessions and total daily training load					Weekly load	
			HR (beats·min^−1^)	HR (%HR_max_)	TRIMP (AU)	RPE (AU)	sRPE (AU)	sRPE training only	sRPE training/ games combined
Coyne et al. [39]	Professional	All players	–	–	–	–	–	–	4588 ± 1597
Anderson et al. [51]	Collegiate	All players	–	–	–	–	530 ± NP^c^	–	–
Cruz et al. [52]	Representative	All players	–	–	–	–	521 ± NP^a,c^	–	1584 ± 237
Ghali et al. [50]	Club	All players	–	–	–	–	–	–	1215 ± NP
Kraft et al. [44]^d^	Collegiate	All players	–	–	313 ± 112	–	711 ± 282^c^	–	–
Lastella et al. [46]	Representative	All players	–	–	–	–	726 ± 456^a,e^	–	–
Lukonaitienė et al. [45]^f^	Representative	All players—U18	–	–	305 ± 172	–	943 ± 437^a,g^	–	–
	Representative	All players—U20	–	–	215 ± 109	–	617 ± 328^a,g^	–	–
Lupo et al. [42]^h^	Representative	All players	–	–	195 ± 57	–	523 ± 122^c^	–	–
Nunes et al. [53]	Professional	All players	–	–	–	–	–	5527 ± 1912	–
Otaegi and Los Arcos [48]	Club	All players—U15	–	–	–	2.7 ± 0.2	213 ± 54^i^	–	879 ± 140
	Club	All players—U16	–	–	–	2.9 ± 0.2	235 ± 39^i^	–	1073 ± 260
Paulauskas et al. [47]	Professional	All players	–	–	–	–	–	1722 ± 369	2505 ± 466
Piedra et al. [54]	Professional	All players	–	–	–	5.4 ± 2.2	534 ± 224^c^	–	–
Reina et al. [35]	Club	All players	147 ± NP	79 ± NP	–	–	–	–	–
Sansone et al. [49]	Semi-professional	All players	–	–	–	–	428 ± 114^j^	–	1561 ± 177

Definitions for the external load variables are described in Table 2. *AvF*_*net*_ average net force, *PL* PlayerLoad™, *AU* Arbitrary units, *IMA* Inertial movement analysis, *n* Number of events, *external TRIMP* External training impulse, *Endash (–)* Not collected, *U18* Under 18 years of age, *U20* Under 20 years of age, *NP* Not provided, *HR* Heart rate, internal *TRIMP* Internal training impulse, *RPE* Rating of perceived exertion, *sRPE* session-RPE load calculated as RPE * session duration (min), *U15* Under 15 years of age, *U16* Under 16 years of age, ^a^Values reported are indicative of total daily training load, ^b^Value is indicative of player load not PlayerLoad™ as described in Table 2, ^c^Did not report how training duration was determined, ^d^Used a modified Summated-Heart-Rate-Zones method to calculate internal TRIMP (described in Table 2), ^e^Session duration was measured from the start to the end of training inclusive of warm-up/down, ^f^Used Banister’s method to calculate internal TRIMP (described in Table 2), ^g^Session duration was measured from the start to the end of training excluding warm-up only, ^h^Used Edwards’ Summated-Heart-Rate-Zones method to calculate internal TRIMP (described in Table 2), ^i^Session duration was measured from the start to the end of training excluding stretching exercises, and ^j^Session duration was measured from the start to the end of training excluding warm-down only

#### Total Daily Training Load

The total daily training loads (sum of all training sessions within a day) experienced in female players are shown in Table 6. Total daily PL, sRPE, and Edwards’ TRIMP were reported across multiple studies or player samples examining representative players. PL ranged from 706 ± 295 AU [45] to 816 ± 333 AU [45] in representative players with a weighted mean of 761 ± 314 AU [45]. sRPE ranged from 521 ± NP AU [52] to 943 ± 437 AU [45] in representative players. A weighted mean could not be calculated for total daily sRPE in representative players given the seasonal phase monitored varied across studies and the approach to measure session duration was not clearly identified across all studies. Internal TRIMP ranged from 215 ± 109 AU [45] to 305 ± 172 AU [45] in representative players with a weighted mean of 260 ± 141 AU [45].

#### Weekly Training Load and Weekly Training and Game Load

The weekly training and weekly training and game loads reported in female basketball players are presented in Table 6. Multiple studies reported weekly training sRPE and weekly training and game sRPE. Weekly training sRPE was only reported in professional players and ranged from 1722 ± 369 AU [47] to 5527 ± 1912 AU [53]. A weighted mean could not be calculated for weekly training sRPE in professional players given the seasonal phase monitored and adopted RPE scale varied across studies, and the approach to measure session duration was not clearly identified across all studies. Weekly training and game sRPE ranged from 879 ± 140 AU in club players [48] to 2505 ± 466 AU in professional players [47]. Weekly training and game sRPE was reported in multiple studies examining club players, ranging from 879 ± 140 AU [48] to 1215 ± NP AU [50] with a weighted mean of 1161 ± NP AU across studies [48, 50].

### External Load During Games Only

#### Activity Distance

Distances covered performing various basketball-specific activities during basketball games reported in female players are presented in Table 7. The absolute total distance covered, relative total distance covered, and distances covered performing sprinting activity in games were reported across multiple studies. The absolute total distance covered during live game time ranged from 2513 ± 1300 m in representative players [34] to 5125 ± 314 m in semi-professional players [32]. The absolute total distance covered during total game time ranged from 2238 ± NP m in representative players [33] to 7039 ± 446 m in semi-professional players [32], while the relative total distance covered ranged from 93 ± 3 m·min^−1^ in high-school players [57] to 117 ± NP m·min^−1^ in representative players [33]. The total distance covered performing sprinting activity during live game time ranged from 14 ± 24 m in representative players [34] to 925 ± 184 m in semi-professional players [32].

**Table 7:** Distance covered (m) for various activities during basketball games in female basketball players according to playing level and playing position

Study	Playing level	Sample	Activity category							Total	
			Stand/walk	Jog	Run	Sprint	Low shuffle	High shuffle	Dribble	Absolute (m)	Relative (m·min^−1^)
Oba and Okuda^a^ [57]	High-school	All players	–	–	–	–	–	–	–	5587 ± 171	93 ± 3
	Collegiate	All players	–	–	–	–	–	–	–	5576 ± 202	100 ± 4
	Professional	All players	–	–	–	–	–	–	–	6177 ± 264	94 ± 4
Portes et al.^b^ [34]	Representative	All players	–	–	–	14 ± 24	–	–	–	2513 ± 1300	–
		BC	–	–	–	13 ± 22	–	–	–	2175 ± 1227	–
		FC	–	–	–	16 ± 26	–	–	–	2802 ± 1300	–
Reina et al.^a^ [33]	Representative	All players	401/616	504 ± NP	492 ± NP	224 ± NP	–	–	–	2238 ± NP	117 ± NP
		BC	326/495	425 ± NP	395 ± NP	174 ± NP	–	–	–	1816 ± NP	115 ± NP
		FC	439/676	543 ± NP	541 ± NP	249 ± NP	–	–	–	2449 ± NP	118 ± NP
Scanlan et al.^b^ [32]	Semi-professional	All players	456 ± 20	1517 ± 93	1850 ± 13	925 ± 184	70 ± 19	55 ± 14	342 ± 44	5125 ± 314	130 ± 8
		BC	410 ± 9^c^	1558 ± 80	1744 ± 52^c^	857 ± 163	75 ± 14	61 ± 8	738 ± 64^c^	5443 ± 238	136 ± 6
		FC	485 ± 27	1491 ± 89	1924 ± 26	970 ± 226	68 ± 34	51 ± 22	76 ± 41	5064 ± 348	127 ± 9
		All players	–	–	–	–	–	–	–	7039 ± 446^b^	–
		BC	–	–	–	–	–	–	–	7371 ± 391^b^	–
		FC	–	–	–	–	–	–	–	6817 ± 487^b^	–

Definitions for the activity categories reported are described in Table 2. *Endash (–)* Not collected, *BC* Backcourt (point guards and shooting guards), *FC* Frontcourt (power forwards, small forwards, and centers), / Standing and walking data were separately provided without standard deviations rather than grouped together, *NP* Not provided, ^a^Values reported according to total time (see Table 3), ^b^Values reported according to live time (see Table 3), ^c^Significantly (*p* < 0.05) different from FC

#### Activity Frequency

The frequency of basketball-specific activities performed during basketball games reported in female players is presented in Table 8. During live game time, absolute movement frequency (*n*) ranged from 576 ± 110 movements in professional players [30] to 1750 ± 186 movements in semi-professional players [32], while relative movement frequency ranged from 21 ± NP movements·min^−1^ in collegiate players [29] to 44 ± NP movements·min^−1^ in semi-professional players [32]. The absolute frequencies performed during live game time were also reported across multiple studies for various basketball-specific activities including: standing/walking: 151 ± 26 in collegiate players [29] to 436 ± 44 in semi-professional players [32]; jogging: 67 ± 17 in collegiate players [29] to 551 ± 67 in semi-professional players [32]; running: 33 ± NP in professional players [31] to 295 ± 41 in semi-professional players [32]; sprinting: 6 ± NP in professional players [31] to 108 ± 20 in semi-professional players [32]; low-intensity shuffling: 41 ± 5 in semi-professional players [32] to 127 ± NP in professional players [31]; moderate-intensity shuffling: 33 ± NP in professional players [31] to 123 ± 45 in collegiate players [29]; high-intensity shuffling: 8 ± NP in professional players [31] to 58 ± 19 in collegiate players [29]; and jumping: 19 ± 10 in professional players [30] to 43 ± 6 in semi-professional players [32]. A weighted mean could only be calculated for jumping during live game time in professional players with values ranging from 19 ± 10 [30] to 30 ± NP [31] and a weighted mean of 28 ± NP across studies [30, 31].

**Table 8:** Frequency (*n*) of various activities performed during basketball games in female basketball players according to playing level and playing position

Study	Playing level	Sample	Activity category											All activity combined	
			Stand/walk	Jog	Run	Sprint	Low shuffle	Moderate shuffle	High shuffle	Jump	Dribble	Upper body	Steps	Absolute (n)	Relative (n∙min^−1^)
Conte et al.^a^ [30]	Professional	All players	205 ± 42	73 ± 20	63 ± 16	44 ± 15	91 ± 23	56 ± 20	25 ± 10	19 ± 10	–	–	–	576 ± 110	23 ± NP
Delextrat et al.^a^ [31]	Professional	All players	177/58	113 ± NP	33 ± NP	6 ± NP	127 ± NP	33 ± NP	8 ± NP	30 ± NP	–	–	–	–	24 ± NP
		BC	178/63	120 ± NP	39 ± NP	9 ± NP	121 ± NP	47 ± NP	15 ± NP	24 ± NP	–	–	–	690 ± NP	25 ± NP
		FC	179/57	109 ± NP	28 ± NP	4 ± NP	131 ± NP	26 ± NP	5 ± NP	34 ± NP	–	–	–	658 ± NP	24 ± NP
Matthew and Delextrat^a^ [29]	Collegiate	All players	151 ± 26	67 ± 17	52 ± 19	49 ± 17	117 ± 14	123 ± 45	58 ± 19	35 ± 11	–	–	–	652 ± 128	21 ± NP
Ransdell et al.^b^ [36]	Collegiate	All players	–	–	–	–	–	–	–	90 ± 33	–	–	–	–	–
		BC	–	–	–	–	–	–	–	94 ± 35^c^	–	–	–	–	–
		FC	–	–	–	–	–	–	–	86 ± 31	–	–	–	–	–
Reina et al.^b^ [35]	Club	All players	–	–	–	–	–	–	–	8 ± 5	–	–	2323 ± NP	–	–
Reina et al.^b^ [33]	Representative	BC	–	–	–	21 ± NP	–	–	–	–	–	–	–	–	–
		FC	–	–	–	29 ± NP	–	–	–	–	–	–	–	–	–
Scanlan et al.^a^ [32]	Semi-professional	All players	436 ± 44	551 ± 67	295 ± 41	108 ± 20	41 ± 5	–	22 ± 5	43 ± 6	34 ± 2	220 ± 18	–	1750 ± 186	44 ± NP
		BC	412 ± 31	547 ± 49	295 ± 33	97 ± 21	48 ± 1	–	25 ± 4	43 ± 5	59 ± 4^c^	223 ± 31	–	1749 ± 158	44 ± NP
		FC	452 ± 54	553 ± 82	297 ± 52	117 ± 22	37 ± 9	–	20 ± 7	41 ± 6	18 ± 5	217 ± 10	–	1752 ± 212	44 ± NP

Definitions for the activity categories reported are described in Table 2. No SD was provided for Delextrat et al. [31] as values were calculated by multiplying the reported relative frequency values by the total live time reported. *Endash (–)* Not collected, *NP* not provided, *BC* Backcourt players (point guards and shooting guards), *FC* = Frontcourt players (power forwards, small forwards, and centers), / Standing and walking data were separately provided without standard deviations rather than grouped together, ^a^Values reported according to live time (see Table 3), ^b^Values reported according to total time (see Table 3), ^c^Significantly (*p* < 0.05) different from FC

#### Activity Duration

Percentages of live and total game time performing different basketball-specific activities during basketball games reported in female players are presented in Table 9. The percentages of live game time spent performing various basketball-specific activities during games were reported across multiple studies including: standing/walking: 35.7 ± NP% in semi-professional players [32] to 50.2 ± 5.5% in professional players [30]; jogging: 11.7 ± 2.9% in professional players [30] to 35.6 ± NP% in semi-professional players [32]; running: 4.9 ± 2.6% in professional players [31] to 16.7 ± NP% in semi-professional players [32]; sprinting: 0.6 ± 0.6% [31] to 5.2 ± 1.8% [30] in professional players; low-intensity shuffling: 3.1 ± NP% in semi-professional players [32] to 16.8 ± 8.8% in professional players [31]; moderate-intensity shuffling: 2.8 ± 2.6% [31] to 6.5 ± 2.4% [30] in professional players; high-intensity shuffling: 0.7 ± NP% in semi-professional [32] and professional players [31] to 2.7 ± 1.4% in professional players [30]; and jumping: 0.6 ± 0.3% [30] to 2.3 ± 1.3% [31] in professional players. Weighted means could only be calculated for activity duration in professional players across studies for standing/walking = 42.0 ± NP %, jogging = 21.3 ± 9.5%, running = 6.7 ± 4.3%, sprinting = 1.6 ± 2.2%, low-intensity shuffling = 15.3 ± 8.3%, moderate-intensity shuffling = 3.6 ± 3.0%, high-intensity shuffling = 1.1 ± 1.6%, and jumping = 1.9 ± 1.4% [30, 31].

**Table 9:** Percentage (%) of basketball game-play performing various activities in female basketball players according to playing level and playing position

Study	Playing level	Sample	Activity category								
			Stand/walk	Jog	Run	Sprint	Low shuffle	Moderate shuffle	High shuffle	Jump	Dribble
Conte et al.^a^ [30]	Professional	All players	50.2 ± 5.5	11.7 ± 2.9	13.1 ± 2.4	5.2 ± 1.8	10.0 ± 2.7	6.5 ± 2.4	2.7 ± 1.4	0.6 ± 0.3	–
Delextrat et al.^a^ [31]	Professional	All players	39.7 ± NP	24.0 ± 9.0	4.9 ± 2.6	0.6 ± 0.6	16.8 ± 8.8	2.8 ± 2.6	0.7 ± 1.4	2.3 ± 1.3	–
		BC	38.5 ± NP	24.6 ± 9.6	5.5 ± 2.1	1.1 ± 0.8	17.4 ± 9.4	4.0 ± 2.9	1.2 ± 2.2	1.5 ± 0.7	–
		FC	40.3 ± NP	23.8 ± 7.9	4.5 ± 2.6	0.4 ± 0.5	16.3 ± 8.5	2.2 ± 2.3	0.4 ± 0.6	2.7 ± 1.1	–
Reina et al.^b^ [33]	Representative	All players	18.1/27.1	22.3 ± NP	21.7 ± NP	10.9 ± NP	–	–	–	–	–
		BC	18.7/26.8	22.6 ± NP	21.5 ± NP	10.4 ± NP	–	–	–	–	–
		FC	17.8/27.2	22.2 ± NP	21.8 ± NP	11.1 ± NP	–	–	–	–	–
Scanlan et al.^a^ [32]	Semi-professional	All players	35.7 ± NP	35.6 ± NP	16.7 ± NP	4.1 ± NP	3.1 ± NP	–	0.7 ± NP	–	4.1 ± NP
		BC	31.0 ± NP^c^	36.2 ± NP	16.0 ± NP^c^	3.7 ± NP	3.6 ± NP	–	0.9 ± NP	–	8.6 ± NP^c^
		FC	38.8 ± NP	35.1 ± NP	17.2 ± NP	4.3 ± NP	2.8 ± NP	–	0.7 ± NP	–	1.1 ± NP

Definitions for the activity categories reported are described in Table 2. *Endash (–)* Not collected, *BC* Backcourt (point guards and shooting guards), *FC* Frontcourt (power forwards, small forwards, and centers), / Standing and walking data were separately provided without standard deviations rather than grouped together, *NP* Not provided, ^a^Values reported according to live time (see Table 3), ^b^Values reported according to total time (see Table 3), ^c^Significantly (*p* < 0.05) different from FC

#### Microsensor Variables

External load variables obtained via microsensors during basketball games reported in female players are presented in Table 10. Relative player load and average net force during total game time were reported across multiple studies. Relative player load was reported in multiple studies examining club players and ranged from 1.2 ± 0.2 AU·min^−1^ [35] to 2.8 ± NP AU·min^−1^ [37] with a weighted mean of 1.9 ± NP across studies [35, 37]. Average net force ranged from 240 ± NP N in semi-professional players [41] to 259 ± NP N in professional players [41].

**Table 10:** External load variables obtained using accelerometers, inertial measurement units, or local positioning systems in female basketball players according to playing level and playing position during basketball game-play

Study	Playing level	Sample	AvF_net_	PlayerLoad™/Player load		Accelerations		Decelerations		High-intensity IMA events	Speed	
			N	Absolute (AU)	Relative (AU∙min^−1^)	Absolute (n)	Relative (n∙min^−1^)	Absolute (n)	Relative (n∙min^−1^)	Absolute (n)	Average (m·s^−1^)	Maximum (m·s^−1^)
Oba and Okuda^a^ [57]	High-school	All players	–	–	–	–	–	–	–	–	–	7.0 ± 0.5
	Collegiate	All players	–	–	–	–	–	–	–	–	-	7.4 ± 0.3
	Professional	All players	–	–	–	–	–	–	–	–	–	8.0 ± 0.5
Palmer et al.^b^ [41]	Semi-professional	All players	240 ± NP	–	–	–	–	–	–	–	–	–
	Professional	All players	259 ± NP	–	–	–	–	–	–	–	–	–
Portes et al.^a^ [34]	Representative	All players	–	39 ± 21	1.0 ± 0.4	370 ± 285	9 ± 5	273 ± 239	7 ± 4	–	–	–
		BC	–	35 ± 21	0.9 ± 0.4	331 ± 256	9 ± 5	230 ± 199	6 ± 3	–	–	–
		FC	–	43 ± 21	1.0 ± 0.4	404 ± 331	9 ± 5	310 ± 265	7 ± 4	–	–	–
Ransdell et al.^b^ [36]	Collegiate	All players	–	656 ± 173	7.1 ± 1.2	–	–	–	–	52 ± 19	–	–
		BC	–	677 ± 171^c^	7.5 ± 1.2^c^	–	–	–	–	51 ± 20^c^	–	–
		FC	–	637 ± 173	6.7 ± 1.1	–	–	–	–	53 ± 17	–	–
Reina et al.^b^ [35]	Club	All players	–	39 ± 20	1.2 ± 0.2	–	–	–	–	–	–	–
Reina et al. [60]	Representative	All players	–	–	–	–	4 ± NP	–	3 ± NP	–	–	–
		BC	–	–	–	–	4 ± NP	–	4 ± NP	–	–	–
		FC	–	–	–	–	4 ± NP	–	3 ± NP	–	–	–
Reina et al.^b^ [37]	Club	All players	–	–	2.8 ± NP	–	–	–	–	–	–	–
		BC	–	–	3.1 ± NP	–	–	–	–	–	–	–
		FC	–	–	2.6 ± NP	–	–	–	–	–	–	–
Reina et al.^b^ [33]	Representative	All players	–	–	–	–	–	–	–	–	1.5 ± NP	5.4 ± NP
		BC	–	–	–	–	–	–	–	–	1.5 ± NP	5.3 ± NP
		FC	–	–	–	–	–	–	–	–	1.5 ± NP	5.5 ± NP

Definitions for each external load variable reported are described in Table 2. Values reported by Ransdell et al. [36] are indicative of PlayerLoad™ (Catapult Innovations; Melbourne, Australia). *AvF*_*net*_ Average net force, *IMA* Inertial movement analysis, *AU* Arbitrary units, *n* Number of events, *Endash (–) *Not collected, *NP* Not provided, *BC* Backcourt players (point guards and shooting guards), *FC* Frontcourt players (power forwards, small forwards, and centers), ^a^Values reported according to live time (see Table 3), ^b^Values reported according to total time (see Table 3), ^c^Significantly (*p* < 0.05) different from FC

### Internal Load During Games Only

#### Internal Load Variables

Internal load variables obtained during basketball games reported in female players are presented in Table 11. Absolute HR, relative HR, and the percentages of live time spent above and below 85% HR_peak_ during games were reported across multiple studies. The mean absolute HR reported during total game time ranged from 136 ± 6 beats·min^−1^ in semi-professional players [32] to 172 ± 8 beats∙min^−1^ in professional players [58] with relative HR (%HR_peak_) ranging from 69 ± 3% HR_peak_ in semi-professional players [32] to 90 ± 14% HR_peak_ in collegiate players [59]. The mean absolute HR reported during live game time ranged from 162 ± 3 beats·min^−1^ in semi-professional players [32] to 186 ± 6 beats·min^−1^ in professional players [56] with relative HR ranging from 82 ± 1% HR_peak_ in semi-professional players [32] to 95 ± NP% HR_peak_ in professional players [56]. The percentage of total game time spent above 85% HR_peak_ ranged from 76 ± 16% in professional players [58] to 80 ± NP% in collegiate players [29], while the percentage of total game time spent below 85% HR_peak_ ranged from 20 ± NP% in collegiate players [29] to 24 ± 16% in professional players [58]. Absolute HR was reported in multiple studies examining club players during total game time and ranged from 147 ± 4 beats·min^−1^ [35] to 169 ± NP beats·min^−1^ [37] with a weighted mean of 157 ± NP beats·min^−1^ across studies [35, 37]. Absolute HR was also reported in multiple studies examining collegiate players during total game time and ranged from 149 ± 2 beats·min^−1^ [43] to 165 ± 9 beats·min^−1^ [29]. A weighted mean could not be calculated for absolute HR during total game time in collegiate players given the approach to measure session duration varied across studies, and the minimum exposure time set for including player data was not clearly identified across all studies. Relative HR was reported in multiple studies examining club players during total game time and ranged from 79 ± 8% HR_peak_ [35] to 85 ± NP% HR_peak_ [37]. A weighted mean could not be calculated for relative HR during total game time in club players given the approach to measure session duration, minimum exposure time set for including player data, and method to identify HR_peak_ (to measure session intensity) were not clearly identified across all studies. Relative HR was also reported in multiple studies examining collegiate players during total game time and ranged from 89 ± 4% HR_peak_ [29] to 90 ± 14% HR_peak_ [59]. A weighted mean could not be calculated for relative HR during total game time in collegiate players given the approach to measure session duration varied across studies and the minimum exposure time set for including player data was not clearly identified across all studies. In turn, absolute and relative HR was only reported in professional players during live game time and ranged from 176 ± 10 beats∙min^−1^ (89 ± 4% HR_peak_) [55] to 186 ± 6 beats·min^−1^ (95 ± NP% HR_peak_) [56]. A weighted mean could not be calculated for absolute and relative HR during live game time in professional players given the approach to measure session duration and method to identify HR_peak_ (to measure session intensity) varied across studies, and the minimum exposure time set for including player data was not clearly identified across all studies.

**Table 11:** Absolute and relative heart rate (HR), blood lactate concentration (BLa), rating of perceived exertion (RPE), and session rating of perceived exertion load (sRPE) responses to basketball game-play in female basketball players according to playing level and playing position

Study	Playing level	Comparison group	Variables reported across entire games			
			BLa (mmol·L^−1^)	RPE (AU)	sRPE (AU)	TRIMP (AU)
Matthew and Delextrat [29]	Collegiate	All players	5.2 ± 2.7	–	–	–
Otaegi and Los Arcos^a^ [48]	Club	All players—U15	–	3.6 ± 1.2	316 ± 115	–
		All players—U16	–	4.5 ± 1.0	378 ± 96	–
Rodriquez-Alonso et al. [56]	Professional	All players	5.3 ± 1.9	–	–	–
		BC	6.2 ± 1.5	–	–	–
		FC	4.9 ± 1.9	–	–	–
	Professional	All players	4.9 ± 2.0	–	–	–
		BC	6.5 ± 2.1	–	–	–
		FC	4.5 ± 1.9	–	–	–
Sanders et al.^a^ [43]	Collegiate	All players	–	–	–	320 ± 77
		BC	–	–	–	281 ± 88
		FC	–	–	–	336 ± 73
Scanlan et al. [32]	Semi-professional	All players	3.7 ± 1.4	–	–	–
		BC	3.8 ± 1.0	–	–	–
		FC	3.7 ± 1.6	–	–	–

Study	Playing level	Comparison group	Variables reported relative to total time			
			Absolute HR (beats∙min^−1^)	Relative HR (%HR_peak_)	% time spent < 85% HR_peak_	% time spent > 85% HR_peak_
Matthew and Delextrat [29]	Collegiate	All players	165 ± 9	89 ± 4	20 ± NP	80 ± NP
Reina et al. [35]	Club	All players	147 ± 4	79 ± 8	–	–
Reina et al. [37]	Club	All players	169 ± NP	85 ± NP	–	–
		BC	173 ± NP	87 ± NP	–	–
		FC	168 ± NP	84 ± NP	–	–
Sanders et al. [43]	Collegiate	All players	149 ± 2	–	–	–
		BC	135 ± 13	–	–	–
		FC	143 ± 11	–	–	–
Sanders et al. [59]	Collegiate	All players	–	90 ± 14	–	–
		BC	–	93 ± 8	–	–
		FC	–	86 ± 13	–	–
Scanlan et al. [32]	Semi-professional	All players	136 ± 6	69 ± 3	–	–
		BC	142 ± 10	72 ± 5	–	–
		FC	132 ± 6	67 ± 3	–	–
Vencúrik and Nykodým [58]	Professional	All players	172 ± 8	88 ± 3	24 ± 16	76 ± 16
		BC	170 ± 9	88 ± 4	27 ± 21	73 ± 21
		FC	173 ± 8	88 ± 4	24 ± 14	76 ± 14

Study	Playing level	Comparison group	Variables reported relative to live time			
			Absolute HR (b∙min^−1^)	Relative HR (%HR_peak_)	% time spent < 85% HR_peak_	% time spent > 85% HR_peak_
Matthew and Delextrat [29]	Collegiate	All players	170 ± 8	93 ± 3	7 ± NP	93 ± NP
Rodriquez-Alonso et al. [56]	Professional	All players	175 ± 13	91 ± NP	–	–
		BC	186 ± 5	93 ± NP	–	–
		FC	171 ± 12	90 ± NP	–	–
	Professional	All players	186 ± 6	95 ± NP	–	–
		BC	190 ± 3	96 ± NP	–	–
		FC	183 ± 5	94 ± NP	–	–
Scanlan et al. [32]	Semi-professional	All players	162 ± 3	82 ± 1	–	–
		BC	161 ± 9	82 ± 5	–	–
		FC	163 ± 5	83 ± 3	–	–
Vala et al. [55]	Professional	All players—Div I	183 ± 13	92 ± 5	–	–
		BC	175 ± 9	91 ± 6	–	–
		FC	187 ± NP	92 ± NP	–	–
	Professional	All players—Div II	176 ± 10	89 ± 4	–	–
		BC	183 ± 7	90 ± 4	–	–
		FC	172 ± NP	88 ± NP	–	–

*AU* Arbitrary units, *BC* Backcourt players (point guards and shooting guards), *FC* Frontcourt players (power forwards, small forwards, and centers), *NP* Not provided, *TRIMP* Training impulse, ^a^Values reported according to total time (see Table 3) and used a modified Summated-Heart-Rate-Zones method to calculate internal training impulse (TRIMP) (described in Table 2)

sRPE during total game time was also reported across multiple player samples and ranged from 316 ± 115 AU in U15 club players [48] to 378 ± 96 AU in U16 club players [48] with a weighted mean of 352 ± 104 AU [48]. Additionally, BLa was reported across multiple studies and ranged from 3.7 ± 1.4 mmol·L^−1^ in semi-professional players [32] to 5.3 ± 1.9 mmol·L^−1^ in professional players [56]. An apparent difference emerged for BLa between playing positions with higher BLa in backcourt players compared to frontcourt players (5.2 ± 1.9 mmol·L^−1^ vs. 4.4 ± 1.8 mmol·L^−1^) [32, 56].

## Discussion

Our review is the first to comprehensively collate research reporting the external and internal loads experienced during training and games in female basketball players. Despite 32 studies being conducted on this topic, surprisingly few load variables have been measured following consistent methodologies across studies. The non-standardized measurement of external and internal load variables across studies prevented the ability to draw definitive conclusions regarding the typical training and game loads experienced by female basketball players according to playing level and position for most variables. From a practical perspective, inconsistencies in the literature regarding the seasonal phase monitored, minimum exposure time set for including player data, approach to measure session duration, approach to measure session intensity, and duration of monitoring periods make it difficult for basketball coaches and researchers to select appropriate load variables and follow uniform procedures when monitoring female basketball players. To address this issue, we provide recommendations to enhance the methodological rigor and promote greater consistency in approaches adopted across future studies investigating external and internal loads in female basketball players.

### External and Internal Loads During Training

#### Individual Training Sessions

Weighted means for loads experienced during individual training sessions in female basketball players could only be calculated for average net force in professional players and sRPE in club and representative players. Specifically, average net force ranged from 272 ± NP N [41] to 293 ± 40 N in professional players [40] with a weighted mean of 281 ± NP N [40, 41]. In this regard, the highest average net force value for individual training sessions was indicative of longitudinal monitoring across 18 training sessions [40], while the lowest value reported for individual training sessions was indicative of longitudinal monitoring across 54 training sessions [41]. Analyzing fewer total training sessions may skew results as acute monitoring periods likely misrepresent the average net force experienced across the wider season due to factors that could allow coaches to administer increased training loads across acute timeframes (e.g., more days between games, less or no travel for games, fewer games played). Furthermore, while data from these studies [40, 41] were collected during different basketball seasons, the outcomes reported were indicative of the same professional basketball team. Consequently, the inclusion of new players, progression of physical (e.g., lean muscle mass and percentage body fat) or physiological (e.g., speed and anaerobic capacity) attributes in players, and potential changes in training approaches or coaching staff between seasons may have contributed to the variation in average net force reported across studies.

Multiple studies reported sRPE during individual training sessions in club and representative players. In this regard, 1 of the 2 studies [44] investigating sRPE in collegiate players failed to report a measure of intensity (i.e., RPE), while both studies neglected to report training duration [44, 51]. Furthermore, 1 of the 2 studies [44] investigating sRPE in collegiate players failed to specify the RPE scale used, preventing us from calculating a weighted mean. Accordingly, we recommend future studies should aim to clearly report the constituent data comprising sRPE values (i.e., RPE scores and session durations) as well as identify the specific RPE scale used to allow for meaningful comparisons in sRPE data across studies examining female basketball players.

Given the amount of published research exploring load monitoring in female basketball players, the fact that average net force and sRPE were the only load variables reported during individual training sessions across multiple studies highlights a lack of attention given to understanding how training is prescribed at the session level as opposed to longer periods (e.g., weekly, seasonal phase). Furthermore, based on the available data, it is unclear how the loads experienced during individual training sessions vary between female players competing at different playing levels or occupying different playing positions. We recommend future studies quantifying weekly external and internal training load to report the load experienced during individual training sessions to allow basketball coaches to better understand how training volume and intensity are altered between weekly microcycles across the season.

#### Total Daily Training Load

We were only able to calculate a weighted mean for loads accumulated across all training sessions completed in a day in female basketball players for total daily PL and internal TRIMP in representative players. In this regard, total daily training PL ranged from 706 ± 295 AU in U20 representative players [45] to 816 ± 333 AU in U18 representative players [45] with a weighted mean of 761 ± 314 AU [45] across age groups, while internal TRIMP ranged from 215 ± 109 AU in U20 representative players [45] to 305 ± 172 AU in U18 representative players [45] with a weighted mean of 260 ± 141 AU [45] across age groups. Given the available total daily training PL and internal TRIMP data were reported in the same study for different player samples (i.e., U18 and U20 players) during intensive training camps, the variance in daily load is likely explained by the different training configurations prescribed for each age group rather than methodological inconsistencies. In this way, U20 representative players completed fewer daily training sessions than U18 representative players during the training camp (U18: 14 out of 21 days had 2 training sessions; U20: 8 out of 18 days had 2 training sessions [45]), reducing their activity exposure to lower the average accumulated daily loads experienced.

#### Weekly Training Load and Weekly Training and Game Load

Although multiple studies reported weekly training sRPE in professional female basketball players, differences in the seasonal phase monitored, RPE scale used, and monitoring period duration prevented weighted means from being calculated across studies [47, 53]. Specifically, Nunes et al. [53] observed 19 professional basketball players from the Brazilian National Team during a 12-week preparatory training camp, while Paulauskas et al. [47] examined 29 professional basketball players from the first division Lithuanian Women’s Basketball League during a 24-week in-season period. In this regard, preparation periods typically involve longer and/or more frequent training sessions at higher intensities (i.e., overloading) than the in-season to promote positive adaptations in preparation for competition [53]. In turn, lower training loads are typically encountered during the in-season compared to preparatory training periods among basketball teams to optimize player readiness for games [64]. Consequently, the weekly loads experienced by female basketball players are likely dependent on the seasonal phase monitored, which should be clearly described in future studies and considered when interpreting reported data. Additionally, 1 of the 2 studies [47] investigating weekly training sRPE in professional players failed to clearly identify the RPE scale used. Given the absolute sRPE value derived when monitoring loads is dependent on the RPE scale used [65], calculating a weighted mean across studies not clearly specifying the RPE scale adopted might yield misleading findings.

Weekly training and game sRPE was only reported across multiple studies in club players ranging from 879 ± 140 AU [48] to 1215 ± NP AU [50] with a weighted mean of 1161 ± NP AU [48, 50]. The variation in weekly training and game sRPE reported is likely explained by the monitoring periods utilized across studies. Specifically, Ghali et al. [50] collected data across a 1-week period at some point in the season that was not identified, while Otaegi and Los Arcos [48] collected data across a 9-week in-season period. The longer monitoring period utilized by Otaegi and Los Arcos [48] likely encompassed week-to-week fluctuations in training and game sRPE experienced by players whereby training was likely adjusted dependent upon in-season factors, such as game scheduling and travel requirements. In turn, the shorter monitoring period utilized by Ghali et al. [50] was likely not representative of the typical weekly training and game loads encountered across the entire season given week-to-week fluctuations in sRPE as high as 47% have been reported across the in-season phase in professional female basketball players [47]. As such, future basketball research should aim to maximize the monitoring period duration to best understand the typical weekly training and game loads imposed on female players.

The lack of studies reporting weekly training and game loads in semi-professional and professional players is surprising as basketball teams competing at these levels likely possess more resources (e.g., finances, staff expertise) than teams competing at lower levels to implement comprehensive player monitoring systems. Furthermore, load data are essential to permit evidence-based decisions that optimize the training and game stimuli encountered, readiness for games, and risk of maladaptive responses in players competing in semi-professional and professional leagues given the arduous demands they face [39, 49]. The deficiency in studies reporting weekly training or weekly training and game loads in semi-professional and professional female basketball players currently limits the ability to comprehensively compare data across playing levels, which can be used in benchmarking processes when transitioning players to higher playing levels.

### External and Internal Loads During Games

#### External load

Despite multiple studies reporting activity distances, frequencies, and durations in female basketball players across different playing levels and positions, weighted means could not be calculated due to several methodological variations across studies. First, this review identified 9 studies reporting movement frequency, duration, and distance covered during basketball games using different technological approaches (video-based TMA, microsensors, and LPS) along with different software packages (LabVIEW, Dartfish, sPRO, SVIVO, Openfield, WIMU, Dynamic Image Analysis System, and LINCE multiplatform analysis). While the use of various technologies across studies is inevitable due to prohibitive factors such as cost and the long-term availability of equipment, the use of various software packages likely introduces variation in the acquired data given undisclosed proprietary algorithms and filtering processes are used in some packages. Second, the number (i.e., 1–4), brand (i.e., Sony, Basler, JVC, DKH, or not reported), positioning (e.g., placement around court, distance from court, height above court), and recording frequency (i.e., 7.5 Hz, 25 Hz, 30 Hz, or not reported) of cameras used for video-based TMA varied between studies. These camera-related variations across studies likely impact the data given the accuracy of vision-based systems is affected by the distances between cameras and players, camera angles, and lens type in the cameras. Third, studies categorized movement and intensities using various methods (irrespective of monitoring technology), including subjective movement categories and intensities identified using frame-by-frame playback of video [29–31, 66], objective speed zones with no justification [33, 34, 60], and objective speed zones [32] based on research examining other court-based team sports [67]. The use of various methods to categorize activity movement and intensity likely impacted the reported outcomes as the criteria used to define a given activity (e.g., sprint) were inherently inconsistent across studies. For example, one study [32] categorized running activity as multidirectional movement performed at 3.1–7 m·s^−1^, whereas two studies categorized sprinting activity as forwards or backward movement performed at > 4 m∙s^−1^ [33] or > 5.8 m∙s^−1^ [34]. Consequently, methodological inconsistencies between studies impeded the ability to definitively determine the typical activity demands experienced during female basketball games according to playing level and playing position.

#### Internal load

We were only able to draw conclusions for BLa given it was the only variable reported across multiple studies. BLa is used as an indicator of energy re-synthesis from rapid glycolysis [29, 32]. In turn, BLa ranged from 3.7 ± 1.4 mmol·L^−1^ in semi-professional players [32] to 5.3 ± 1.9 mmol·L^−1^ in professional players [56] during games. The BLa values reported highlight the utilization of the rapid glycolytic energy pathway in executing game activities in female basketball players [29, 56]. As such, implementation of anaerobic conditioning drills incorporating prolonged and repeated high-intensity actions [68] is essential to improve tolerance of high BLa and enhance lactate threshold markers in female players. In this regard, aerobic conditioning is also critical to maximize lactate clearance and improve phosphocreatine regeneration during recovery periods between repeated high-intensity activities across games [69]. Moreover, given multiple studies [32, 56] reported BLa in female basketball players during games according to playing position, we were able to calculate and compare weighted means for backcourt and frontcourt players. In this regard, a higher BLa was apparent in backcourt players compared to frontcourt players (5.2 ± 1.9 mmol·L^−1^ vs. 4.4 ± 1.8 mmol·L^−1^) [32, 56]. These position-specific variations in BLa might be explained by the strategic roles typically performed in each position during games. Specifically, backcourt players typically undertake frequent intense cutting movements to create space for open perimeter shots and defend opposing perimeter players cutting to receive the ball [70]. Moreover, backcourt players are more likely to be involved in fast breaks as they initiate steals [71] or leak out when transitioning into offense as well as pursue opposing backcourt players when transitioning to defense. These intense movements performed frequently across games by backcourt players likely increase the reliance on rapid glycolysis for energy re-synthesis [72, 73] compared to frontcourt players who are typically positioned closer to the basket on offense and defense.

While multiple studies reported the absolute and relative HR of club and collegiate female basketball players as well as absolute and relative HR according to playing position during games, some key methodological variations across studies impeded the ability to calculate weighted means and draw definitive conclusions. First, ‘total time’ was inconsistently defined across studies, with studies defining ‘total time’ as the time during which the player was on the court including stoppages in play but not time-outs or breaks, including all stoppages in play (i.e., free-throws, out-of-bounds, and time-outs) but not breaks [29, 37, 43], or including all breaks and stoppages in play [59]. Given rest periods between quarters and halves as well as during stoppages in play enable extra opportunities for recovery and reductions in HR, the inconsistent inclusion or exclusion of breaks and stoppages in play would have altered the outcomes reported across studies. Second, HR_peak_ was determined using various methods, including peak responses taken during an incremental treadmill test [29, 43, 59], peak responses taken during basketball training sessions [35], and peak responses taken during a 20-m shuttle run [32], or the method to determine HR_peak_ was not reported [37]. Third, playing time criteria for including HR data from players were not specified [35, 37, 56, 58] or varied across studies with some studies using player data regardless of total playing time [35, 37, 59], if players accumulated ≥ 3 min of live playing time in any given quarter and ≥ 10 min of live playing time for the entire game [43], or if players accumulated ≥ 25 min of live playing time for the entire game [29]. The use of different playing time criteria for data inclusion likely impacted the reported outcomes as shorter playing times are expected to elicit higher HR values during live game time but lower HR values during total game time compared to longer playing times. For example, during live game time, short spurts of activity are likely to produce rapid spikes in HR as a result of an increased oxygen deficit, while the inclusion of stoppages such as time-outs, out-of-bounds, and free-throws is likely to decrease the HR response during total game time due to increased recovery opportunities.

### Limitations and Future Directions

Our review provides important information for basketball coaches and performance staff regarding the external and internal loads experienced during training and games in female basketball players; however, there are limitations that must be considered when applying the reported findings. On a positive note, the limitations encountered in conducting our review have brought much needed attention to the methodological inconsistencies across published research examining load monitoring in female basketball players, permitting us to develop recommendations aimed at improving the quality of future research in the field.

First, given the limited number of studies reporting external and internal loads in players competing in the same basketball league, we were unable to aggregate data according to basketball league. The game rules and competition format (e.g., game scheduling, game durations) are inconsistent across many basketball leagues, which may impact the external and internal game loads experienced by players and should be taken into account when interpreting the data presented.

Second, defining the type of players involved in studies is critical for understanding differences in external and internal loads between playing levels and playing positions, which is essential to develop training targets for basketball coaches. However, descriptors used to classify playing level and playing position were inconsistent across the included studies, which limited the ability to compare findings between studies. For example, the term ‘elite’ was used to describe several playing samples ranging from youth players in U14 club teams, collegiate players, and professional players. Regarding playing position, some studies categorized players into two playing positions as either frontcourt and backcourt [32, 40, 61] or guards and posts [36], while other studies categorized players into three (i.e., guards, forwards, and centers [33, 34, 43, 55, 56, 58–60]) or five (i.e., point guard, shooting guard, small forward, power forward, and centers [31, 37]) playing positions, but with different categorical criteria for each position. Therefore, to allow for comparisons between studies, playing level data were recategorized from lowest to highest as follows: club, high-school, collegiate, representative (trained athletes selected into a representative team), semi-professional (some players are full-time/contracted athletes), or professional (all players are full-time, contracted athletes), while positional data were recategorized into backcourt and frontcourt. Future research should seek to establish a consensus regarding the categorization of playing level and playing position in basketball research to better allow for comparisons between studies.

Third, external loads reported in our review were derived from various technologies, including video-based TMA, LPS, and microsensors (containing triaxial accelerometers, gyroscopes, magnetometers or a combination of these instruments). In this regard, the criteria (i.e., speed or intensity zones) used to distinguish between movement intensities and the formulae or algorithm used to calculate external load variables (i.e., Catapult PL vs. player load) were inconsistent across studies. Using various criteria (e.g., speed cut points) to distinguish between movements performed during training and games is likely to over- or under-estimate the external intensities being performed and prohibit meaningful comparisons in findings across studies. Consequently, expert consensus should be sought to establish cut points for basketball-specific speed or intensity zones with different approaches to monitor external load to allow for consistent and accurate classification of movements or intensities in future basketball research.

Fourth, training and game durations were determined inconsistently across studies, with some studies not specifying the methods adopted to measure session duration. This limitation should be considered when interpreting the data reported in our review. In turn, future basketball research should be transparent and detailed in describing the procedures used to measure training and game duration, with separate reporting of warm-up and cool-down components alongside other session components being advocated [74].

Finally, data collection was predominantly reported across acute periods in the included studies (12 ± 9 weeks). While the duration of data collection may vary based on the specified research aims across studies, the acute time periods used in most studies may produce skewed results due to the impact of factors that can directly influence training prescription and game demands such as game scheduling [75, 76]. Furthermore, most studies (67%) monitored players during the in-season phase only. The use of a single seasonal phase limits the applicability of the reported outcomes in practice as training load fluctuates across seasonal phases due to changes in training approaches and the physiological capacities of players [77, 78]. As such, we recommend future research to examine longer monitoring periods as well as different seasonal phases to gain a comprehensive understanding of the external and internal loads experienced in female basketball players during training and games across the annual plan.

## Conclusions

Our review is the first to comprehensively collate research reporting external and internal load variables during training and games in female basketball players. Despite the amount of published research conducted in female basketball players, discrepancies in the methods utilized to measure common load variables across studies and a lack of published data for specific playing levels and positions limited our ability to make definitive conclusions regarding the external and internal loads typically experienced during training and games. However, the inconsistent measurement of load variables and variations in methodologies across studies will likely persist until key load variables as well as standardized methodologies are established and promoted among researchers in the field and a position stand is released by an established organization. It is essential that standardized approaches are established for: (1) categorizing playing level and position; (2) determining when to include player data in analyses (e.g., minimum exposure time); (3) measuring session duration (e.g., total time, live time, session components); and (4) measuring session intensity (e.g., consistent RPE scales, intensity zone cut points) in future female basketball research to permit meaningful interpretation and comparisons of load monitoring data across studies. Moreover, it is vital that future female basketball studies are conducted across different playing levels and monitor players longitudinally across different seasonal phases while reporting load data across varying timeframes (e.g., individual sessions, weekly, monthly) to better identify how player demands fluctuate and understand the periodization approaches adopted in different teams.

## Data Availability

Not applicable.
